# Racial and Ethnic Diversity in Medical School Admissions in Canada

**DOI:** 10.1001/jamanetworkopen.2023.24194

**Published:** 2023-07-19

**Authors:** Ye Bin Shin, Amanda Stojcevski, Taylore Dupuis-Miller, Amrit Kirpalani

**Affiliations:** 1 Department of Paediatrics, Schulich School of Medicine and Dentistry, Western University, London, Ontario, Canada; 2 Division of Nephrology, Children’s Hospital, London Health Sciences Centre, London, Ontario, Canada

## Abstract

**Importance:**

Medical schools promote admission pathways as well as diversity and inclusivity to prospective students who are underrepresented in medicine (UIM) primarily via their websites. Research from organizational structures supports the use of instrumental values (which focus on behavior and core beliefs), rather than terminal values (which focus on the end goal), to promote diversity.

**Objective:**

To characterize the use of instrumental and terminal values to promote institutional diversity and inclusivity on Canadian medical school websites.

**Design, Setting, and Participants:**

This qualitative study analyzed the contents and themes of Canadian medical school websites based on a theoretical value framework and an organizational diversity management framework. A literature review was conducted to explore the use of instrumental and terminal values in promoting diversity and inclusivity, and a codebook was created that outlined the characteristics of these values. The content of all application pathway websites for UIM applicants to all Canadian medical schools was coded between July and August 2022.

**Main Outcomes and Measures:**

Use of terminal vs instrumental values across the UIM-targeted admission websites of medical schools.

**Results:**

Twenty-two websites for 17 Canadian medical schools were included. Content on most of these websites promoted diversity in response to government policies or public accountability and often supported a dominant culture while treating diversity as an exception. The use of terminal rather than instrumental values was also more prominent on websites that targeted Indigenous prospective students compared with Black prospective students and applicants with lower socioeconomic status (11 [50%] websites vs 5 [23%] and 4 [18%] websites). Instrumental values, although seldom used, promoted diversity as a means to improve health care and training for all students in a program.

**Conclusions and Relevance:**

Results of this qualitative study suggested that medical schools in Canada promoted diversity and inclusivity to prospective students primarily as an end goal rather than as a core value. Medical schools should highlight the benefits of diverse lived experiences and reinforce their commitment to diversity and inclusion by aligning their website content with their ongoing work in this area.

## Key Points

**Question:**

Do medical schools promote diversity as a core belief (instrumental value) or as an end goal (terminal value)?

**Findings:**

In this qualitative study of 22 websites for 17 Canadian medical schools, most institutions promoted diversity primarily as an end goal rather than a core value, often in response to government policies or public accountability, and enforced a dominant culture while portraying diversity as an exception.

**Meaning:**

Findings of this study suggest that medical schools need to highlight the benefits of diverse lived experiences and reinforce their commitment to diversity and inclusion.

## Teaser

This qualitative study evaluates how racial diversity and inclusivity are promoted in online admissions material of Canadian medical schools.

## Introduction

Medical schools commonly offer admission pathways for applicants who are underrepresented in medicine (UIM). However, matriculants from these pathways frequently report facing discrimination from their peers.^1^ Previous studies of applicant perceptions have revealed prominent misconceptions about the quality of UIM matriculants and the lack of understanding of and appreciation for diversity and representation in medical schools.^2^ While initiatives to increase admission of UIM applicants are critical, they have not always been accompanied by organizational systems that maximize the potential advantages of diversity. For these prospective students, although matriculation is important, their individual success may be threatened when institutions lack supportive infrastructure.^3^

For medical schools, the opportunities to promote diversity and inclusivity are countless; however, to reach prospective students, schools often rely on their websites as the first point of contact and one of their largest platforms. Admission pathways for UIM applicants in particular are primarily advertised via the school websites. As a platform, the website is a public-facing expression of the institutional mission statement and commitment to diversity and inclusivity, which may reflect its values.

On the surface, any expression in favor of diversity and inclusivity may seem to be worthwhile for an institution looking to support these principles. However, research within diversity management systems has revealed that the type of values promoted may affect the level of acceptance of diversity within an organization.^4^ The theoretical framework of Rokeach defines value as “an enduring belief that a specific mode of conduct or end-state of existence is personally or socially preferable to an opposite or converse mode of conduct or end-state of existence.”^5^^(p5)^ There are 2 types of values: instrumental values, which guide behavior to achieve a desirable end goal, and terminal values, which refer to the end goal itself. Olsen and Martins,^4^ in researching the role of instrumental and terminal values in organizational management, contended that organizations that emphasize instrumental values of diversity will demonstrate greater acceptance and appreciation for diversity within the group compared with organizations that emphasize diversity as merely a terminal value. That is, institutions focusing on why diversity is important may expect increased workforce diversification and greater embodiment of diversity within the workforce. This argument has been supported by studies in the business sector regarding diversity hiring programs.^6,7,8^ These studies found that companies with instrumental values experienced greater levels of engagement from both minoritized and nonminoritized employees than companies with terminal values, in which there were greater levels of animosity and skepticism from minoritized and nonminoritized employees.^6,7,8^

Adopting this organizational diversity management framework,^4^ we explored Canadian medical schools’ promotion of diversity and inclusion to prospective students, specifically admissions websites that serve as a large applicant-facing platform for UIM groups. We conducted a descriptive content analysis to characterize the use of instrumental and terminal values to promote institutional diversity and inclusivity on Canadian medical school websites. In the context of this study, instrumental values were defined as the pursuit of diversity with intention to leverage it as a resource for an increasingly diverse population. Terminal values, in contrast, were defined as the end goal of simply achieving statistical diversity.

## Methods

In accordance with Western University protocol and Tri-Council Policy Statement Article 2.2, this qualitative study was exempt from ethics board approval because it did not pose any risk to or adversely affect the welfare of any individuals. We followed the Standards for Reporting Qualitative Research (SRQR) reporting guideline.^9^

### Theoretical Value Framework and Codebook Development

We adopted the Rokeach^5^ theoretical framework of values. With this framework, we conducted an extensive literature review, exploring the use of instrumental and terminal values in promoting diversity and inclusivity. Due to the paucity of diversity management research in medicine or medical education, we drew from the published literature in the business sector.^6,7,8,10,11,12^

Following this extensive literature review, we prepared a codebook outlining the characteristics of instrumental and terminal values along with codes corresponding to the setting of medical school admission (eAppendix in Supplement 1). We further adopted the values lens in agreement with the organizational diversity management framework of Olsen et al,^4^ which proposed that instrumental values are more likely to foster acceptance of diversity than terminal values.

### Data Collection, Coding, and Reflexivity

Between June and July 2022, we reviewed the websites of all Canadian medical schools, including English-language and French-language schools, and iteratively refined the study’s inclusion criteria for UIM-targeted web pages. While the definition of UIM is not uniform and is continuously being revised in the literature, we sought to capture admission pathway web pages that were aimed at diversification of the incoming class, specifically targeting communities that are historically UIM in Canada. In this conceptual framework, we included admission pathway web pages for Black and Indigenous applicants and those with a lower socioeconomic background.

Race and ethnicity as well as socioeconomic status data were not collected as there were no participants in the study; the data analyzed were from the websites. Data and information from these web pages were collected using NCapture with NVivo (Lumivero, formerly QSR International).

Between July and August 2022, 2 of us (Y.B.S. and A.S.) deductively coded all content on the websites using the constructed codebook (eAppendix in Supplement 1) on NVivo. Videos on these websites were transcribed and coded as well. Coding was an iterative process, involving regular discussions among researchers, and new codes were added as needed.

Authors and contributors of this study had diverse racial and educational backgrounds. Team members also had varying exposures to the medical field and recognize the importance of diversity in medical education and medicine to better patient care. As individual researchers, we have reflected on the potential implications of our cultural and educational biases for data collection and analysis and acknowledge their influence on data interpretation.

### Statistical Analysis 

Using the coded content of the websites, including videos, we conducted a descriptive content analysis with constant peer comparison to characterize the prominent values of medical schools regarding diversity and inclusivity. We then conducted a thematic analysis with Nvivo to organize the coded content into larger themes and to explore how instrumental and terminal values were used within each of these themes.

## Results

A total of 22 websites for 17 Canadian medical schools were included and analyzed. Use of terminal values vs instrumental values was predominant across the websites. This discrepancy was more pronounced on websites that targeted Indigenous prospective students compared with Black applicants and applicants with lower socioeconomic status (11 [50%] websites vs 5 [23%] and 4 [18%] websites) (Figure). Across all websites, the content was clustered into 4 themes (eFigure in Supplement 1): response, dominant culture, identity focused, and leveraging diversity (Box).

**Figure.**
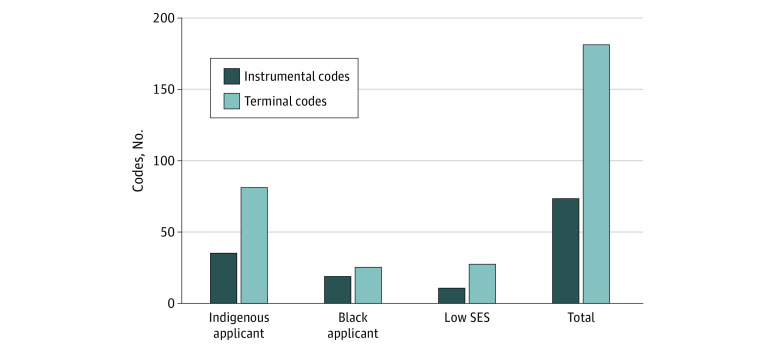
Distribution of Terminal and Instrumental Codes Within Admission Pathways for Underrepresented-in-Medicine Applicants Across Canadian Medical School Websites SES indicates socioeconomic status.

Box. **Themes, Codes, and Values Identified From Medical School Websites Targeted at Applicants Underrepresented in Medicine**

**Response Theme**

- Moral imperative code (terminal value): “As part of our ongoing commitment to diversity, equity, and inclusion, the MD Program … has introduced the Black Applicant Admissions Process.”
- Terminal integration code (terminal value): “We make sure that we have Indigenous file reviewers who are looking at the files of people who are applying through that programme or that stream.”
- Recognition code (terminal value): “Historically, members of the Black community have been underrepresented in both the applicant pool and in undergraduate medical classes.”

**Dominant Culture**

- Terminal integration code (terminal value): “The collection of self-identification data will provide us with information on the diversity of the population.”
- Assimilation code (terminal value): “Your participation could also be mandatory in the Francophone general pathway multiple mini-interviews.” [translated from French]
- Terminal integration code (terminal value): “The difference is that they will have members of the Black, Indigenous and People of Colour (BIPOC) community engage in their file review.”
- Nondiscrimination code (terminal value): “When a candidate has a rating sufficient to be considered within the Quebec regular pathway, their candidacy proceeds according to the admission process specific to each of these two pathways.” [translated from French]

**Identity-Focused Theme**

- Recognition code (terminal value): “Historically, members of the Black community have been underrepresented in both the applicant pool and in undergraduate medical classes.”
- Characteristic code (terminal value): “Applicants who apply through the Indigenous Admission Stream and provide the required documentation may receive a slightly higher context score.”
- Identity-blind code (terminal value): “During evaluation, [applicants] will be ranked against the remainder of the applicant pool.”

**Leveraging Diversity Theme**

- Leverage code (instrumental value): “Our primary obligation is to meet the needs of the populations we serve ... and [ensure that] our students are trained to meet their specific health-care needs.”
- Organizational performance code (instrumental value): “BAAP will undergo the same application process and are held to the same standards as the remainder of the applicant pool.”
- Access code (instrumental value): “... including your current and past experiences/involvement in the Indigenous community ... and your goals for future involvement.”
- Instrumental integration code (instrumental value): “If you have questions, concerns, ideas or a personal story about systemic racism within our [school] community, I encourage you to share your thoughts. Your input will contribute to the [school]’s overall action plan for advocacy and accountability.”

Abbreviation: BAAP, Black applicant admissions process.

### Response Theme

A major concept of the response theme was the promotion of diversity by medical schools in response to government policies or public accountability. This theme consisted of only terminal value codes, such as moral imperative, terminal integration, and recognition.

The recognition code can be perceived as both a terminal and instrumental value. Wording on websites often emphasized the goal of increasing the number of medical school students from the UIM community rather than emphasizing diversity’s added value to the program (eg, “Historically, members of the Black community have been underrepresented in both the applicant pool and in undergraduate medical classes.”). These statements often lacked any explanation of why it is crucial to rectify the current status of underrepresentation and were categorized as promoting the terminal values of diversity. On the other hand, when schools emphasized the need for diversification by recognizing the value and necessity of diverse health care workers, we coded it as promoting the instrumental value of diversity (eg, “We value selecting and promoting a community of health care professionals that reflects the diversity of the Canadian population and the communities we serve.”).

The terminal integration code reflected changes that medical schools implemented uniquely for UIM admission pathways without integrating the same changes into the general stream application process. Such changes included inviting members of the underrepresented communities to be part of the selection process for the UIM admission pathways only (eg, “We make sure that we have Indigenous file reviewers who are looking at the files of people who are applying through that programme or that stream.”). This website content was coded as promoting terminal values as it attempted to integrate underrepresented community members as an exception rather than allowing them to be present throughout the general stream application process. Within the organizational diversity management framework, the response theme emphasized the goal of increasing the number of underrepresented students in medical schools without offering a thorough explanation of the benefits of their lived experience in medicine.

### Dominant Culture Theme

Another theme we identified was dominant culture, wherein medical schools still enforced a dominant culture while making diversity an exception. Codes under this theme were all terminal values, such as terminal integration and assimilation.

As mentioned, the terminal integration code represented changes to integrate diversity into the UIM application process but not into the general (or dominant) stream application process. Websites emphasized the difference of using UIM admission pathways to reduce barriers for UIM applicants without any mention of reducing the barriers to inclusivity within the general stream application process (eg, “The difference is that they will have members of the Black, Indigenous, and People of Colour [BIPOC] community engage in their file review.”). Although such website content highlighted a mechanism to dismantle barriers to matriculation, without further context, this appeared to foster a lack of a shared experience between the general and UIM applicants. Terminal integration could construct a distinction between a dominant culture in the general stream application process and single out the experiences of UIM matriculants as exceptions to existing rules.

The assimilation code described website content suggesting that UIM applicants would be evaluated within the general stream application process in addition to the UIM admission pathway. This content was more subtle as no medical schools strictly required the UIM applicants to culturally assimilate into the dominant culture. However, some programs did emphasize that self-identified UIM applicants may be asked to participate in both UIM and general application process interviews (eg, “You may also be required to participate in the multiple mini-interview in the general applicant stream.” [translated from French]).

This website content suggested that the admission pathway for UIM applicants did not align with that for the dominant culture. Terminal integration codes under the dominant culture theme created a distinct line between applicants, with UIM prospective students being perceived differently from the general applicants at the medical school.

### Identity-Focused Theme

The identity-focused theme represented medical schools’ expressed value of the UIM applicants’ racial and ethnic identity rather than their lived experiences. Codes under this theme were predominantly terminal values, such as characteristic and identity blind.

The characteristic code represented the medical schools’ selection process, wherein, under specific circumstances, UIM applicants were provided with a quantifiable bonus on the basis of identity compared with applicants in the general stream (eg, “Applicants who apply through the Indigenous Admission Stream and provide the required documentation may receive a slightly higher context score.”). Website content in this category presented UIM identity as a measurable criterion in admission, which was viewed as a reinforcement of UIM identity rather than experiences and skill sets as the end goal.

The identity-blind code, on the other hand, represented a selection process wherein the applicants’ background or identity was not factored into the decision process. In contrast to website content that prioritized UIM identity as the end goal, identity-blind content suggested not acknowledging the existing barriers to matriculation facing prospective students from UIM communities nor any potential gains from their lived experiences.

### Leveraging Diversity Theme

A theme composed of uniquely instrumental values was leveraging diversity. This theme supported the idea of improving patient care through the added value of a diverse workforce and lived experiences. Website content within this theme highlighted the potential of diversification to expand the breadth of understanding between patients and health care practitioners about patient-centered and culturally appropriate care. Instrumental value codes, such as leverage and instrumental integration, were grouped within this domain.

The leverage code represented the medical schools’ aim to use diversity as a tool to improve health care for racially and socioeconomically marginalized populations. Wording on websites, such as the following, highlighted how the experiences that shape UIM applicants can fill existing gaps in medicine: “Research shows that increased diversity of medical students leads to unique learning opportunities and to better physicians with a greater ability and understanding of diverse communities. It can also improve access to care for underserved communities and provide better health care to all patients, including minority communities that face unique health challenges.”

Similarly, the instrumental integration code described a medical school’s aim to make changes to the dominant culture based on the voices of members of the UIM community. Medical schools stated that they not only accepted UIM applicants but also supported them in leading transitions (eg, “If you have questions, concerns, ideas or a personal story about systemic racism within our [school] community, I encourage you to share your thoughts. Your input will contribute to the [school]’s overall action plan for advocacy and accountability.” Such support emphasized that diversity was not simply an exception to the norm but rather a necessary and beneficial element in advancing the field of medicine.

## Discussion

Medical schools promoted diversity and inclusivity to prospective students largely as an admirable end goal, placing relatively less emphasis on the desirable behavior or manner of operation that may be gained from diversification of the institution. The prominent portrayal of diversity as a response to a glaring need was based on terminal values. On their websites, medical schools highlighted an inadequacy in the number of UIM matriculants without providing the context for why such underrepresentation exists and the reason it must be rectified. Under the organizational diversity management framework, such messaging is unlikely to foster an appreciation for or acceptance of admission pathways for UIM applicants and may be labeled as tokenism.^4^

It has previously been established that organizational practices that are perceived to be aiming to meet a minimum threshold of acceptance may foster feelings of isolation, loneliness, and low sense of belonging among UIM physicians.^13^ Tokenism itself stems from marginalization and discrimination and only gives the appearance of equality rather than achieving true equality.^13^ The predominant use of terminal values on web pages targeted to UIM applicants highlighted the end goal of diverse classes and ignored the historical injustices associated with the existing underrepresentation of UIM students. Institutions could consider promoting instrumental values by providing the context for inequity on their websites to help UIM and non-UIM applicants understand the behaviors that the institution is seeking to discourage (eg, racism). In particular, medical schools can highlight how their admission values are reflected in their ongoing work and initiatives in diversity and inclusion (ie, bridging the gap between candidate selection and achievement of local educational and health care goals).

The use of terminal values was also prominent in the portrayal of the dominant culture of medical schools. Moreover, UIM prospective students were largely described with the terminal values lens, ascribing their larger identity to their UIM status. Website content appeared to describe the end state of diversity as one in which minoritized individuals are assimilated or integrated, reinforcing the existing power dynamics in medical education. Zaidi et al^14^ have argued that structural racism is inherent to medical education and is largely fostered by socialization of stakeholders in medicine and support for existing social hierarchies. These existing systems also pose a threat to the sense of belonging among social newcomers in medicine, including applicants from racial and ethnic minority groups and from lower socioeconomic backgrounds.^15^ Terminal values, which describe the UIM admission pathway as a means for UIM prospective students to enter and be shaped by the medical infrastructure, do little to dismantle the ingrained power dynamics. However, we did note that instrumental values, when used, were largely seen on websites that aimed to leverage diversity. These websites highlighted the potential for applicants with diverse lived experiences to empower the entire student cohort, as has been seen in cohorts of newcomers to medicine.^15^ The focus on diversity as a means to a valuable end goal is expected to promote acceptance within the class under the organizational diversity management framework of Olsen et al,^4^ and such messaging could be prominently featured on UIM and non-UIM admission pathway web pages.^5^

The results of this study highlight a noticeable deficiency in institutional communication to prospective students of a simple message: diversity is important. However, refinement of UIM-targeted website content should not be considered as a superficial change (ie, embracing tokenism); rather, it should be viewed as an appropriate reflection of the medical school’s commitment to equity, diversity, and inclusivity. It may be better to promote instrumental values by specifying how class diversity is associated with an enhanced training experience of the entire class and improved patient care.^16^ For example, rather than general statements regarding the institutional mission to provide care for underserved communities, specific statements exemplifying the school’s partnership with UIM physicians may be a better way to promote the core instrumental values of diversity (eg, “We aim to train diverse physicians with an understanding that diversity in a class improves the cultural competency of the entire cohort, giving students the skills, knowledge and disposition to compassionately serve communities who are historically and/or presently marginalized, excluded, and divested.”). Initiatives that aim to increase diversity should not be framed as simply a form of reparations for previous injustices but should reinforce the broader benefits of a diverse cohort for medical education and health care overall. Institutions should also avoid an overall rebranding in favor of public acceptance; rather, they should consider involving key stakeholders in determining the specific behaviors they seek to foster among a diversified class and the number of UIM prospective students they need to help them with that mission. Such messaging, when combined with systemic and structural changes from leaders and employees at all levels, is important in creating an inclusive learning environment, allowing UIM and non-UIM trainees to achieve their greatest potential. A focus on why, and not simply what, a medical school wants to achieve with diversity and inclusivity should serve as a foundation of its online presence to prospective students.

### Limitations

This study has several limitations. First, we did not analyze content that was posted on social media platforms, which may also have a substantial reach to potential applicants. Second, the study included data from Canadian medical schools, which may not necessarily be representative of institutions in other countries. Moreover, the UIM admission pathways we analyzed targeted racially and socioeconomically marginalized communities, and due to the lack of pathways for other underrepresented communities (eg, gender and sexual minority groups; people with disabilities), we were unable to include content for these prospective students. Third, while use of terminal values was predominant on medical school websites, the implications of this messaging for current and future applicant cohorts could not be assessed but should be evaluated in a future study. Fourth, applicants’ perception of a medical school’s diversity and inclusivity may be influenced by the reputation of its parent institution. Fifth, we did not analyze the content on the nonadmission web pages of the medical school websites.

## Conclusions

In this qualitative study, Canadian medical schools promoted diversity and inclusivity to applicants primarily as an end goal rather than as a core value. This practice was especially true for Indigenous and Black applicants, the 2 most common UIM groups in Canada. The dominant use of terminal values on medical school websites may be a missed opportunity to educate prospective students on the advantages of a diverse and inclusive medical education. Medical schools should reinforce their commitment to diversity and highlight the benefits of diverse lived experiences by adopting instrumental values and aligning their website content with their ongoing equity, diversity, and inclusion initiatives.

## Notes

Supplement 1. **eAppendix.** Constructed Codebook Outlining Instrumental and Terminal Values **eFigure.** Overview of Themes From Underrepresented in Medicine Admission Web Pages

Supplement 2.
Data Sharing Statement
