# Supplementary material for: Racial and Ethnic Diversity in Medical School Admissions in Canada
Source: JAMA Netw Open. 2023 Jul 19;6(7):e2324194. doi: 10.1001/jamanetworkopen.2023.24194 (PMC10357339; doi:10.1001/jamanetworkopen.2023.24194)
Supplement: Supplement 1. — eAppendix. Constructed Codebook Outlining Instrumental and Terminal Values eFigure. Overview of Themes From Underrepresented in Medicine Admission Web Pages [file jamanetwopen-e2324194-s001.pdf]

Supplemental Online Content

Shin YB, Stojcevski A, Dupuis-Miller T, Kirpalani A. Racial and ethnic diversity in medical school admissions in Canada. *JAMA Netw Open*. 2023;6(7):e2324194. doi:10.1001/jamanetworkopen.2023.24194

eAppendix. Constructed Codebook Outlining Instrumental and Terminal Values

eFigure. Overview of Themes From Underrepresented in Medicine Admission Web Pages

This supplemental material has been provided by the authors to give readers additional information about their work.

© 2023 Shin YB et al. *JAMA Network Open.*

eAppendix. Constructed codebook outlining instrumental and terminal values.

**Instrumental Value**	**Code**	**Terminal Value**	**Code**
Diverse workforce = resource (i.e. the goal is to gain the benefits of a diverse workforce) (*Eg. to better understand diverse patient populations)*	Resource	Diverse workforce itself = objective (i.e. the goal is to have a diverse workforce) (*Eg. to increase more Indigenous applicants)*	Objective
Leveraging diversity for a greater purpose rather than simply aspiring to "maintain" diversity. Leveraging diversity uses diversity for a further goal rather than diversity itself being the goal	Leverage	Social responsibility or commitment (have a responsibility due to social demands or standards)	Imperative
Better understanding of and access to diverse consumers	Access	Equal employment opportunity	Equality
Emphasize recognition, and respect of differences, rather than the suppression of differences (*recognize that a specific group of persons is a minority in medicine and the necessity of diverse healthcare workers)*	Recognition	Emphasize nondiscrimination through establishing norms & policies that discourage reference to demographic differences	Nondiscrimination
Decisions based on organizational criteria (whether the individual will be an	Organizational Performance	Decisions based on individual characteristics (whether the	Characteristic

© 2023 Shin YB et al. *JAMA Network Open.*

asset to the team), (*requiring same MCAT/GPA or other evaluations as the general stream and not altering admission standards)*		individual's characteristics meet their goal), Performance management, reward, and promotion systems more likely to consider physical characteristics (*picking a particular candidate applying through a diversity stream over a candidate in the regular stream)*	
Implement identity-conscious practices (identity-conscious = take group membership into consideration and the merit that can arise from the membership) (*eg. "your lived experience in an Indigenous community serves to benefit the provision of healthcare to Indigenous communities"*	Identity conscious	Implement *both* identity-blind and identity-conscious practices (identity blind = eliminating discrimination by being blind to demographic membership and only considering individual merit) (*eg. not placing value in one's background and experiences but rather simply the skill they can offer)*	Identity blind
Focused on integration (preservation of cultural identities with mutual understanding and respect, encouraging the expression of cultural differences)	Integration	Focused on assimilation (aims to have one dominant culture)	Assimilation

© 2023 Shin YB et al. *JAMA Network Open.*

Instrumental assimilation: recognize diversity as an instrumental value, but they still expect individuals to conform to policies and practices rooted in the dominant culture's norms and values	Instrumental Assimilation	Terminal assimilation: focus on diversity as a terminal value but de-emphasize differences among demographic groups.	Terminal Assimilation
Instrumental integration: recognizes diversity as a means to achieve organizational objectives and encourages organizational members to incorporate their cultural identities to inform business operations	Instrumental Integration	Terminal integration: entails the view that diversity is a desirable end state but does not require nondominant groups to assimilate to the dominant culture is not the right strategy for achieving diversity *(Implements a diversity interview panel only for UIM applicants but not for the general stream. In favor of diversity panel but not for all streams)*	Terminal Integration
Beliefs about desired modes of action eg. being independent. Means to achieve other valued goals	Means	Beliefs about desired end-states eg. freedom, comfortable life. A goal in itself *(End state for the school. Eg. we want to become a diverse community)*	End-State
Favour the utility of the product	Utility	Favour the symbolic meaning of product	Symbolism
Value	Tangible results	Results are less	Intangible results

© 2023 Shin YB et al. *JAMA Network Open.*

tangible/quantifiable attributes (eg. increased consumer sales, greater efficiency)		tangible or intangible [Intangible as in there is no change in performance even after the integration of diverse employees (does not mean there isn't a tangible increase in employee background)]	

© 2023 Shin YB et al. *JAMA Network Open.*

eFigure. Overview of Themes From Underrepresented in Medicine Admission Web Pages

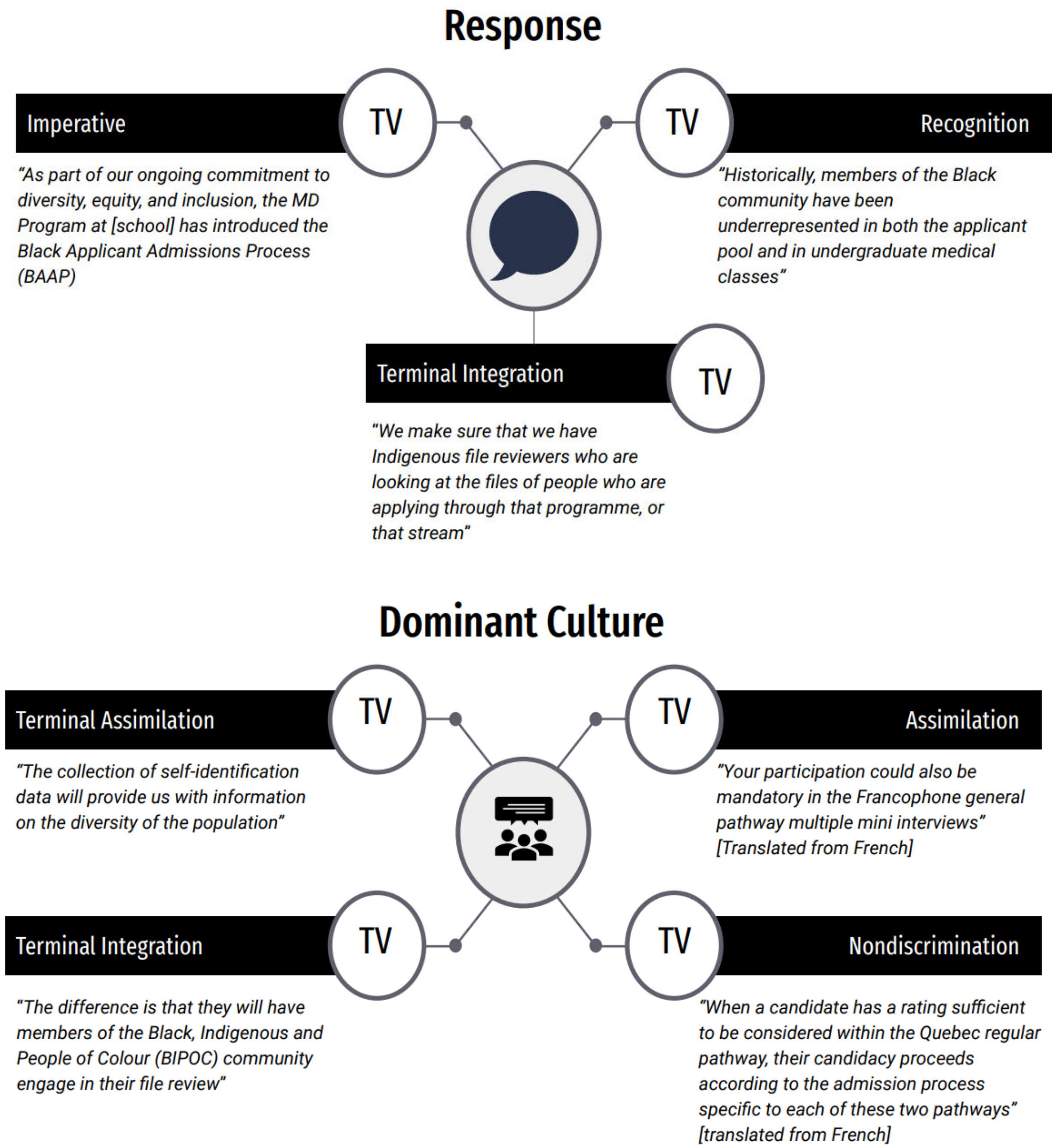

© 2023 Shin YB et al. *JAMA Network Open.*

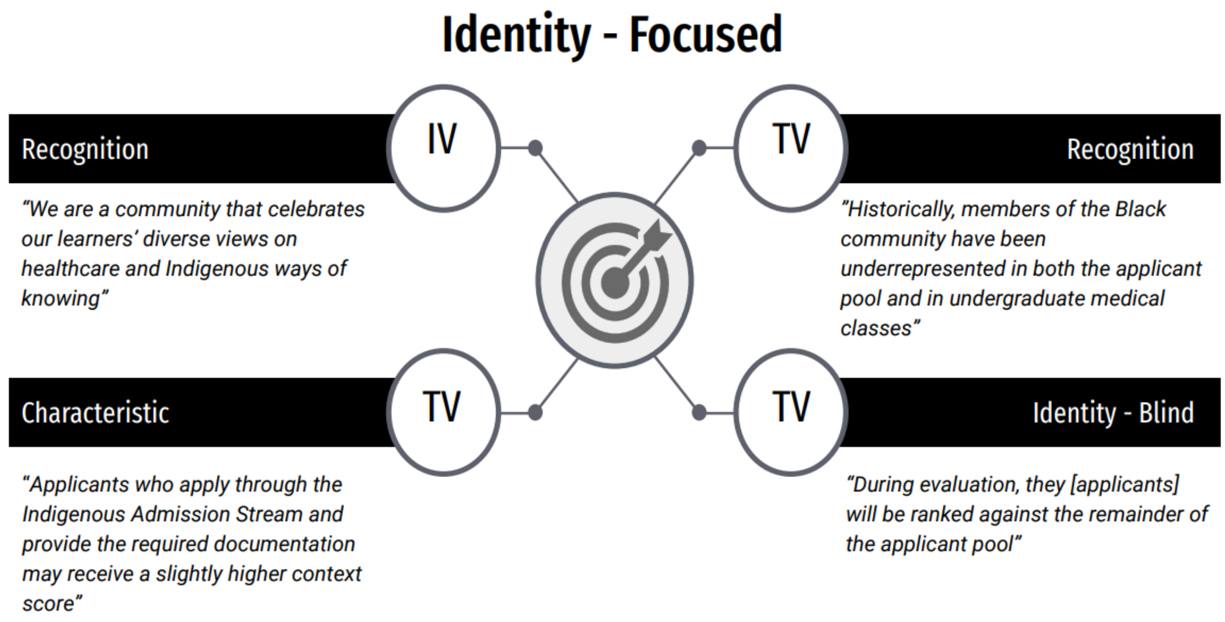

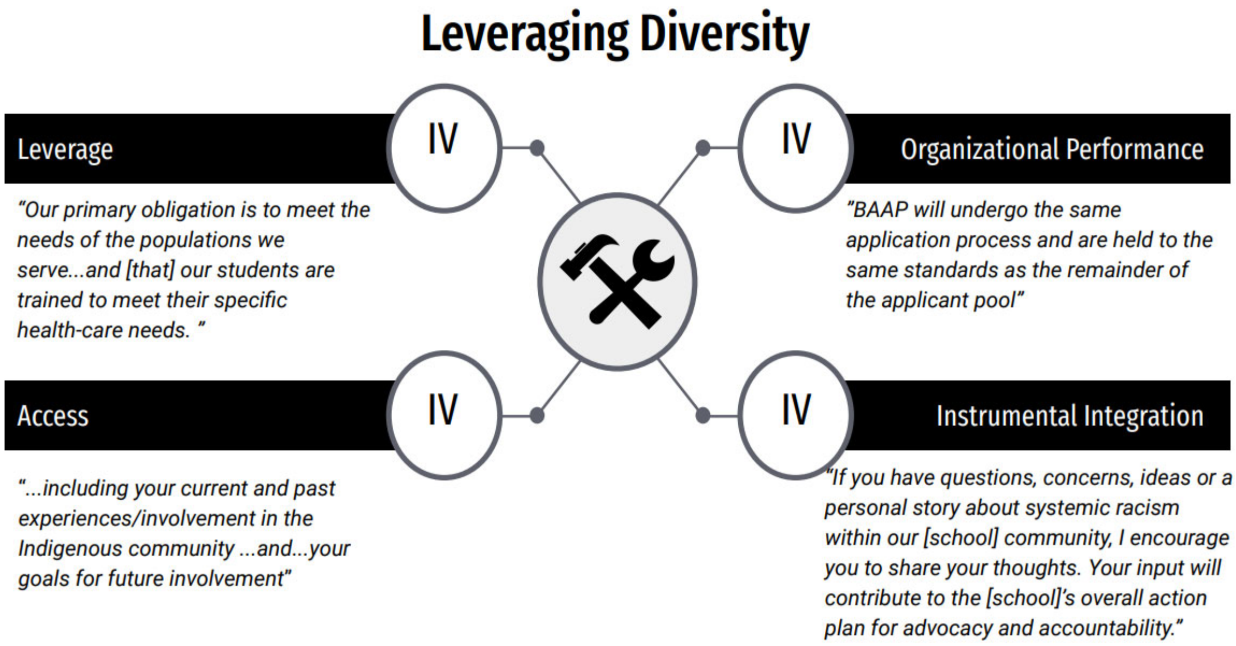

© 2023 Shin YB et al. *JAMA Network Open.*
