# Supplementary material for: Racial and Ethnic Diversity in Medical School Admissions in Canada
Source: JAMA Netw Open. 2023 Jul 19;6(7):e2324194. doi: 10.1001/jamanetworkopen.2023.24194 (PMC10357339; doi:10.1001/jamanetworkopen.2023.24194)
Supplement: Supplement 2. — Data Sharing Statement [file jamanetwopen-e2324194-s002.pdf]

Data Sharing Statement

Shin. Racial and Ethnic Diversity in Medical School Admissions in Canada. *JAMA Netw Open*. Published July 19, 2023. doi:10.1001/jamanetworkopen.2023.24194

Data
Data available: No

Additional Information
Explanation for why data not available: data is publicly available
